# Nanotechnology-driven platforms for extracellular vesicle analysis in tumor immunotherapy

**DOI:** 10.3389/fimmu.2025.1632378

**Published:** 2025-07-30

**Authors:** Rui Chen, Qin Kang, Yudong Ning

**Affiliations:** ^1^ Department of Otolaryngology, Rizhao Central Hospital, Rizhao, China; ^2^ Deparment of Plastic Surgery, Rizhao Dermatology Hospital, Rizhao, China; ^3^ Department of Head and Neck Surgery, National Cancer Center/National Clinical Research Center for Cancer/Cancer Hospital, Chinese Academy of Medical Sciences and Peking Union Medical College, Beijing, China

**Keywords:** immunotherapy, extracellular vesicles, microRNA, protein, liquid biopsy, cancer

## Abstract

Cancer is one of the most challenging diseases, the current treatment of malignant tumors has entered the era of immunotherapy. Immunotherapy has made great progress in the treatment of malignant tumors, but many patients have limited response to treatment. The search for new molecular biomarkers to evaluate the immunotherapy efficacy is particularly important. Liquid biopsy is a non-invasive method that has the advantage of providing real-time disease information to cancer patients. Extracellular vesicles (EVs), released by parental cells, contain important molecules associated with cell growth, proliferation and migration, which are regarded as the targets of liquid biopsy. In addition, EVs also participate in the information communication in tumor immune microenvironment, and are important molecular markers for monitoring the cancer immunotherapy efficacy. In this review, we summarize the challenges of conventional detection methods for EVs, and the advantages of nanotechnology detection of EVs. The important role of EVs in tumor immune microenvironment was discussed and the potential clinical significance of EVs in monitoring and predicting cancer immunotherapy response was summarized.

## 1 Introduction

The emergence of immunotherapy in cancer treatment has brought revolutionary changes to oncology research (1). Immunotherapy aims to reactivate anti-tumor immune cells and overcome tumor immune escape. Tumor immunotherapy, represented by immune checkpoint inhibitors (ICIs) has achieved great success in clinical practice, and can induce long-term regression of some tumors that are difficult to cure by other therapies. Among them, programmed death receptor 1 (PD-1)/programmed death ligand 1 (PD-L1)/inhibitors and cytotoxic T-lymphocyte antigen 4(CTLA-4) are the most important ICIs, and PD-1/PD-L1 inhibitors are most commonly used in clinical practice (2). ICIs, activating the anti-tumor immune response by blocking inhibitory immune signaling has been shown to be effective against a variety of cancers, including non-small cell lung cancer (NSCLC), melanoma, head and neck squamous cell carcinoma (HNSCC), kidney cancer, and breast cancer. However, there are great discrepancies in immune response in different patients and different cancer types, and the underlying mechanism is still far from fully understood (3–8). As a result, current screening methods remain inadequate and reliable predictive biomarkers are urgently needed for personalized clinical management and new treatment strategies.

Tissue biopsy is often used to diagnose malignant tumors, and many biomarkers of immunotherapy are also evaluated by tissue biopsy. In clinical practice, the expression level of PD-L1 is usually detected by immunohistochemistry of tumor biopsy tissue, which is used to screen patients with ICIs (9). Importantly, this tissue-based testing requires an adequate tissue biopsy. For some tumors, there is not enough tumor tissue for molecular testing at the time of diagnosis (10). Tissue biopsies are not only invasive, they may also not fully represent the state of the entire tumor due to the heterogeneity of the tumor (11). During the course of treatment, the immune status of patients is dynamically changing, and repeated and invasive tissue biopsy is not clinically feasible. Such temporal and spatial heterogeneity will inevitably constitute the limitations of tissue biopsy.

Liquid biopsy is a non-invasive method with the advantages of real-time monitoring and minimal damage to cancer patients (12, 13). In the course of immunotherapy, liquid biopsy technology is used to analyze and evaluate the molecular changes in the body of patients after medication, which is conducive to evaluating whether the tumor has progressed (14). Recent approaches to liquid biopsies to identify clinically useful biomarkers have focused on circulating tumor cells (CTCs) and circulating tumor DNA (ctDNA) (15). CTCs have the potential to provide critical information to help develop real-time biomarkers for diagnosis, prognosis, and prediction of treatment response. ctDNA has gained more attraction in clinical practice because its prognostic significance and ability to continuously monitor residual disease during treatment has been demonstrated in several cancer types (16–19). Clinical trials of immunotherapy have further demonstrated the predictive ability of ctDNA as a biomarker associated with survival benefits (15). Nevertheless, CTCs and ctDNA still face a number of challenges that limit their clinical application. CTCs are characterized by short lifespan, low number and concentration, dynamic heterogeneity, often relying on epithelial markers for isolation, and requiring advanced technologies such as microfluidic devices and enrichment strategies to increase sensitivity (20–22). ctDNA only accounts for 0.1-10% of the total circulating free cell DNA (cfDNA) (23).Therefore, analysis of ctDNA often requires a larger blood sample size, and the mutations identified may also reflect non-malignant cells, resulting in false-positive results (20).

Extracellular vesicles (EVs) are a new biomarker of liquid biopsy. EVs isolated from biological liquids are composed of a series of vesicles and nanoparticles with different cell origins, sizes, and concentrations (24). In contrast to ctDNA and CTCs, EVs are superior because of the unique properties as shown below. 1. They are more abundant in biological liquids than CTCs while more informative than ctDNA; 2. EVs can be obtained from variety of biofliuds including blood, cerebrospinal fluid (CSF), urine, etc, other than only blood samples for CTCs and ctDNA; 3. EVs can penetrate through many cell membrane barriers, especially blood-brain barriers, which are critically important for diseases in central nerve system; 4. EVs are relatively stable due to their lipid bilayer and can be stored at -80°C for a relatively long time while preserving their morphology and content (25, 26).

Studies have shown that EVs mediates a variety of biological pathway or mechanism in cancer progression including cell growth, proliferation, and migration, through transferring EV-containing molecules between different cells. Thus cancer-related molecules present in EVs should be biomarkers for the diagnosis and prognosis of cancer patients (27). EVs contain a variety of biomolecules, including DNA, mRNA, microRNA (miRNA), long non-coding RNA (lncRNA), proteins, metabolites, and lipids, which represent the heterogeneity of parental cells, making them an important source of biomarkers (28). Specifically, their changes before and after treatment also show great potential in monitoring therapeutic response (29) to facilitate patient stratification and personalized treatment of cancer patients. In particular, as a key medium of intercellular communication in the tumor microenvironment, EVs could be a critical factors for monitoring of the immunotherapy response (16).

This review summarizes the sources of EVs, the efficient EVs detection methods by nanotechnology, the relationship of EVs with the tumor immune microenvironment, and its application as biomarkers in immunotherapy.

## 2 Sources and detection methods of EVs

### 2.1 The source of EVs

EVs are microvesicles secreted by cells into the extracellular space and various body fluids (30). Microvesicles are vesicles that bud directly from the cell membrane. They are particles and large vesicles with diameters ranging from 50 nanometers (nm) to 1 micrometer (μm). In contrast, exosomes are endoderm - derived vesicles with diameters ranging from 40 to 160 nm (with an average of 100 nm). The formation of exosomes follows a specific intracellular endolysosomal pathway in a step-by-step manner involving several mechanisms. The initial step in exosome formation is that endocytic vesicles arise from the lipid raft domains of the plasma membrane through endocytosis, leading to the intracellular formation of early endosomes. Subsequently, these early endosomes mature into late endosomes in the Golgi complex. During this process, intraluminal vesicles (ILVs) accumulate within the lumen. These vesicles can further accumulate in late endosomes via inward budding or cytosolic sequestration, transforming the late endosomes into multivesicular bodies (MVBs). Eventually, the membrane vesicle biofilms (MVBs) either fuse with the lysosome for degradation or with the plasma membrane, releasing the ILVs into the extracellular space as exosomes (31). Exosomes play a crucial role in cell-to-cell information exchange. They contain molecular information such as phospholipids, proteins, DNA, mRNA, miRNA, and so on. Since exosomes are enveloped by a lipid-bilayer membrane, the RNA information they carry is not easily degraded. This protects the integrity of RNA molecular information and reduces the sampling difficulty. Therefore, the aforementioned characteristics of exosomes determine their significant role in the tumor immune microenvironment.

### 2.2 Challenges and nanotechnology applications for EVs detection

**Figure 1:**
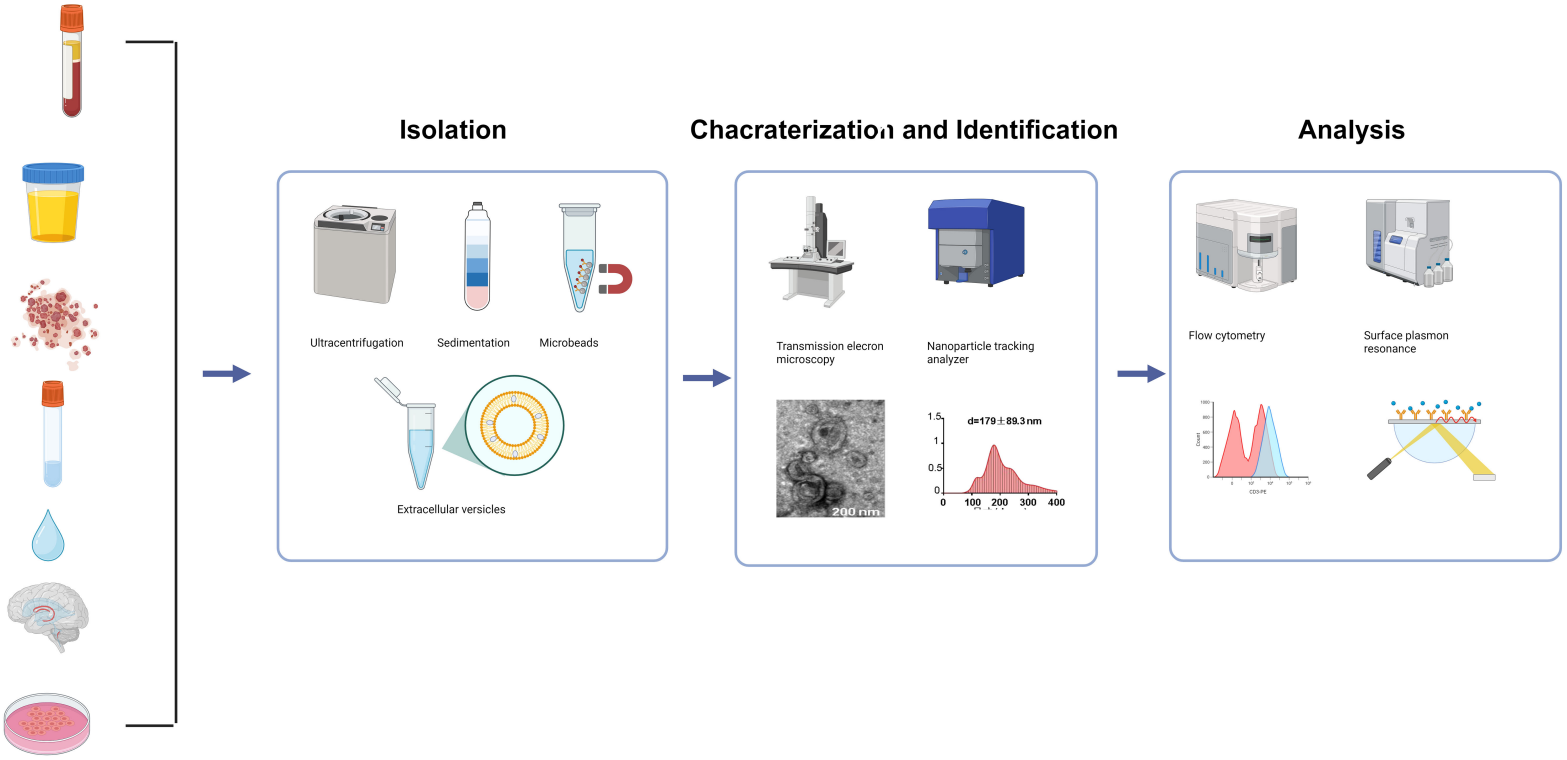
Liquid biopsy based on extracellular vesicles mainly involves the analysis of relevant components in body fluids such as blood, urine, and cerebrospinal fluid. This mainly consists of three processes: isolation, characterization and identification, and analysis.

Detecting and analyzing EVs poses significant technical challenges. Their small size, heterogeneity, and the difficulty of separating them from complex samples, particularly those with complex components like proteins, lipoproteins, and lipids (32),contribute to this. As a result, differentiating EVs from a large number of blood cells and other complex components in blood demands high sensitivity and specificity. Generally, as shown in **Figure 1**, the detection of EVs involves three processes: the isolation of EVs, the characterization and identification of EVs, and the analysis of EVs.

#### 2.2.1 The isolation and enrichment of EVs

**Figure 2:**
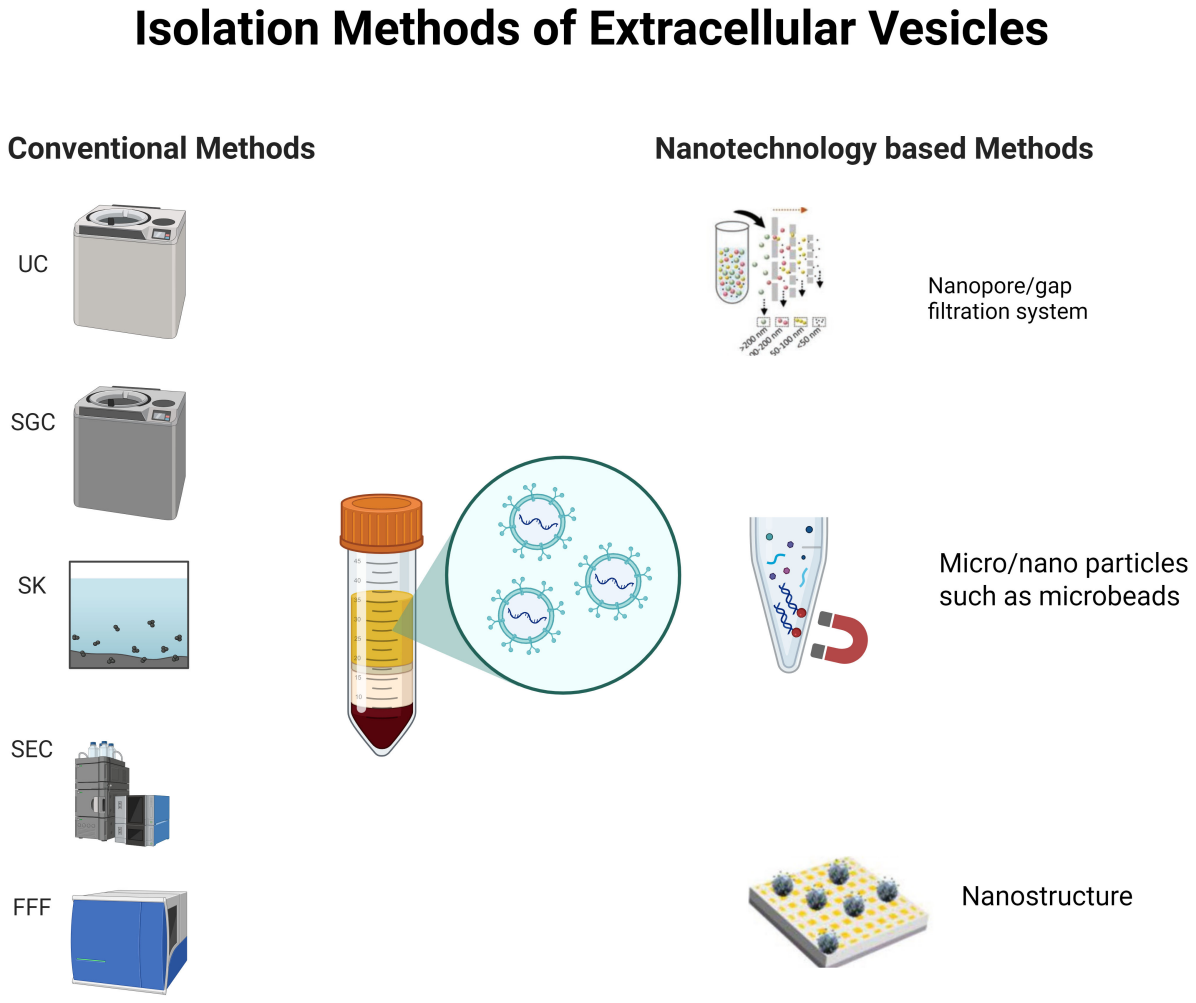
The isolation methods of extracellular vesicles can be categorized into conventional methods and nanotechnology-based methods. Conventional methods include ultracentrifugation (UC), sucrose gradient centrifugation (SGC), sedimentation kits (SK), size-exclusion chromatography (SEC), and field-flow fractionation (FFF). Nanotechnology-based methods encompass the nanopore/gap filtration system, as well as micro/nano particles and nanostructures (33).

As shown in **Figure 2**, the isolation methods of EVs can be categorized into conventional methods and nanotechnology-based methods.

##### 2.2.1.1 Advances and challenges of conventional methods

Based on the mechanism and principle of EVs separation, conventional EV separation methods can essentially be classified into three categories: density-based separation, size-based separation, and immune-based separation. Ultracentrifugation is the most frequently employed method for EVs separation. This method precipitates EVs based on density differences using high-speed centrifugation (100,000×g), serving as the current “gold standard” for EVs isolation. However, it is labor-intensive and prone to contamination by protein aggregates (34). It also serves as the gold standard for EVs isolation and is currently the most commonly used approach in EVs research. Nevertheless, the ultracentrifugation procedure is rather cumbersome, sample handling is complex, and it is impossible to completely eliminate the contamination of aggregates and ribosomal protein particles. Sucrose gradient centrifugation(SGC) is a more stringent method for separating EVs. Building upon UC, this technique further purifies EVs via density gradients, enabling the separation of EV subpopulations with distinct densities (e.g., tumor-derived vs. normal cell-derived EVs). Nevertheless, it is time-consuming (4–6 hours) It builds on ultracentrifugation and exploits the density differences among EVs (3, 4), which is beneficial for further separating vesicles with varying densities (35, 36). Several companies have developed sedimentation kits that utilize polymer coprecipitation strategies to enrich EVs. These agents typically decrease the solubility of EVs by altering the surface properties of the vesicles, thus leading to precipitation. However, these kits are costly, not suitable for large-scale use, and lack specificity in EVs isolation. Moreover, this co-precipitation method also generates a large number of polymer particles that are difficult to distinguish from EVs. As a result, its practical application is restricted. Size-exclusion chromatography(SEC) is a chromatographic separation technique that relies on the gel column method for separation. This method separates EVs by size using a gel column, yielding high-purity EVs. However, it has a slow flow rate (2–3 hours) and struggles to resolve vesicles with similar sizes (37, 38). Molecules flow out of the gel column successively according to their sizes. Field-flow fractionation is another separation technique where a force field is applied perpendicular to the sample flow to differentiate samples with different sizes and molecular weights. Recently, asymmetric flow field flow separation has been applied to EVs isolation (39).

##### 2.2.1.2 Advances of methods by nanotechnology

In this context, nanostructures and nanomaterials exhibit significant advantages in the separation and detection of EVs. This is due to their large surface-to-volume ratio, which can substantially increase the number of binding sites. As a result, it enhances the efficiency of capturing EVs (40–42).Additionally, the nanoscale dimensions of these nanostructures enable the fabrication of substrates featuring densely packed nanostructures. This characteristic offers a valuable opportunity to amplify the local signals emitted by the captured EVs (42).

Nanomaterials and nanostructures utilized for the separation and enrichment of EVs can generally be grouped into three categories: separation relying on physical properties like size, density, deformability, and charge; capture and isolation by means of nanobeads, and enrichment based on nanostructured substrates.

Filtration systems based on nanopores or gaps have been devised to separate and categorize EVs. Leveraging membranes with precise pore sizes (100–1000 nm), this technique enables rapid size-based isolation of EVs subpopulations (e.g., small EVs vs. large EVs), reducing processing time to 30 minutes. Nanopore systems, such as Exodisc (43) and Exotic (44), incorporate a series of nanopore filtration membranes with varying pore sizes. This enables the direct separation of EVs and different-sized subpopulations of EVs from biological samples. Moreover, the nanoporous structure can be adjusted through micro-machining of thin-layer electrode chips. By using surfactant-based electrochemical deposition to grow nanopores, and subsequently applying specific antibodies, it becomes possible to detect and enrich EVs (45).

Separation and enrichment of EVs using micro/nano particles is also a prevalent approach. EVs enrichment with microbeads (either aldehyde-modified or antibody-coated, magnetic or non-magnetic) is frequently employed to boost the detection signal of EVs (46–48). After isolation, the EVs are labeled with fluorescent antibodies for imaging or flow cytometric analysis, or they are cleaved for RNA analysis. An engineered superparamagnetic material, namely gold-loaded iron oxide, which is modified with a universal four-protein antibody, has been developed to capture and detect a large number of EVs (49).

EVs enrichment based on micro/nanoparticles is also integrated with microfluidics for a comprehensive detection and molecular analysis of EVs. The nanostructured substrate offers an excellent opportunity to enhance the capture efficiency and the local binding signal of EVs because of the increased interface. The nanoscale roughness of these substrates augments the physical interaction between EVs and the substrate, thus improving the adhesion of EVs (50). Nanostructured substrates can be readily combined with microfluidic technology, enabling the integrated capture and molecular analysis of EVs (51, 52). Moreover, various releasing strategies can be applied to nanostructured substrates to retrieve the captured EVs and to facilitate the downstream molecular analysis (53, 54). Common nanostructured surfaces for EVs include horizontally aligned nanostructures, such as nanotextured films covering substrates (55)and vertically aligned nanostructures, such as nanowires (56).The high surface-to-volume ratio of nanowires provides more binding sites for interaction with EVs. They can readily work in conjunction with antibodies, peptides, or aptamers to capture EVs. Additionally, they can be modified with reactive materials, such as those with enzymatic or pH reactivity, to release the captured EVs (57). Numerous studies have verified the effectiveness of nanostructure-based and nanotechnology-based EV analysis in the diagnosis, prognostic assessment, and monitoring of various tumors (58).

#### 2.2.2 Characterization and identification of EVs

**Figure 3:**
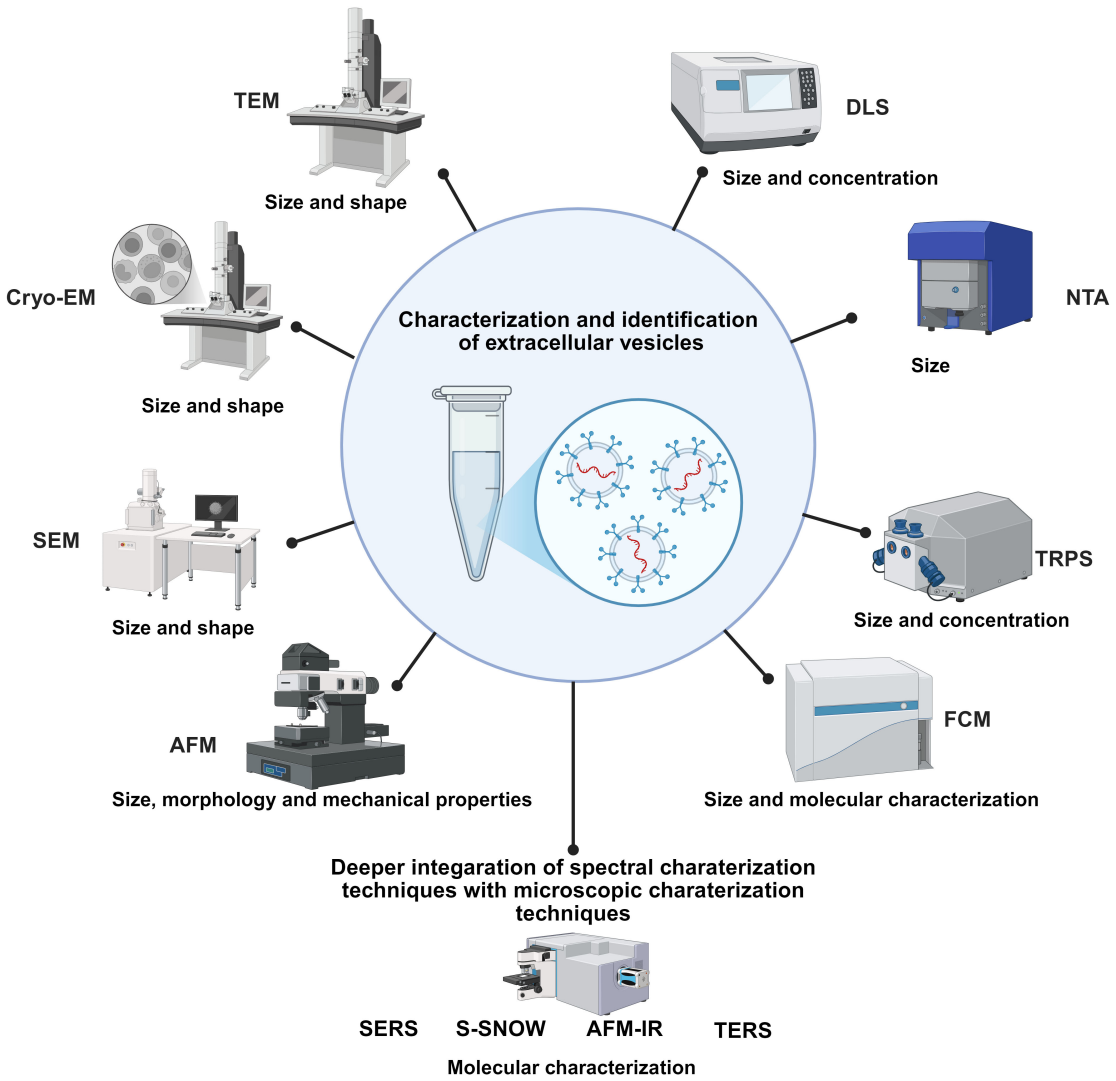
Characterization and identification of extracellular vesicles involve various techniques, namely transmission electron microscopy (TEM), cryogenic electron microscopy (Cryo-EM), scanning electron microscopy (SEM), atomic force microscopy (AFM), nanoparticle tracking analyzer (NTA), dynamic light scattering (DLS), tunable resistance pulse sensing (TRPS), flow cytometry (FCM), surface-enhanced Raman scattering (SERS), tip enhanced Raman spectroscopy (TERS), scattering-type scanning near-field optical microscopy (S-SNOM), and atomic force microscopy-infrared spectroscopy (AFM-IR).

As shown in **Figure 3**, characterization and identification of EVs involve various techniques. In this chapter, we provide a comprehensive review of the technologies for characterization and analysis of EVs.

##### 2.2.2.1 Physical characterization of EVs

Measuring the concentration and size of EVs is a crucial standardization factor in EV research, which contributes to enhancing the repeatability of experimental data. The concentration and size of EVs can be determined through light scattering, resistance pulse sensing, surface plasmon resonance, and numerous other methods with comparable capabilities.

Dynamic light scattering (DLS), also known as photon correlation spectroscopy, is a light scattering technique. It employs a laser beam to measure the size of particles in suspension and, in certain cases, their zeta potential (59). The size measurement range of DLS techniques is generally from as small as 1 nm up to particles larger than a micron (60). Nevertheless, the sample sources suitable for DLS are severely restricted to non-biological ones. Otherwise, some filtration steps are necessary to decrease the heterogeneity of the sample.

Nanoparticle Tracking Analyzer (NTA) is a commonly used approach for assessing particle concentration and size distribution. It works by recording the light spots generated by EVs when they are illuminated by a laser beam, using a microscope camera, and tracking their Brownian motion (61). NTA distinguishes itself from DLS techniques in that it can measure both the light-scattering intensity and the size of individual particles, enabling higher resolution analysis for heterogeneous sample populations. The typical size detection range of NTA is 10–1000 nm, though this value can vary depending on the refractive index (RI) and the signal-to-noise ratio of the sample. Additionally, false signaling is a persistent issue, mainly caused by the aggregation of particles and proteins. This is often why NTA is used in combination with a zeta potential reader to avoid such false signals (62).

Tunable Resistance Pulse Sensing (TRPS) is a novel non-optical technique that is currently being utilized to quantify the size and concentration of EVs (63). This technique makes use of a non-conductive nanopore membrane, through which EVs can pass driven by a current of charged ions. The fundamental principle of this technique is based on the combined action of electrophoretic and convective flow, which are induced by external pressure and an applied voltage. Each EVs passing through the charged pores causes a change in the pore’s resistance. This resistive pulse can be detected as a transient change in the current, which is proportional to the particle volume and concentration (64).Regrettably, this technique has several significant drawbacks. Firstly, it is technically difficult to implement due to the heterogeneous size range of EVs and the problem of pore clogging. Secondly, the calibration of beads in TRPS depends on the buffer components of the sample, which are often unknown when measuring EVs, especially for biological samples (65).

Flow cytometry (FCM) is not only capable of detecting particles in a sample but also of characterizing the structure and morphology of EVs (66). FCM is particularly suitable for reproducible studies of clinical samples. It is a powerful tool that enables the simultaneous multi-parameter analysis of up to thousands of particles per second. Thus, it is an effective method for quantifying, separating, and purifying particles in suspension. However, due to the overlap between the light scattering of particles and background noise, a large number of particles, especially small ones, cannot be characterized by conventional flow cytometry. To overcome these problems, high-resolution FCM, which features higher sensitivity, forward scattering detection, fluorescence amplification, and high-resolution imaging, can effectively distinguish the signals of stained EVs from the background (67).

##### 2.2.2.2 EV topology characterization

Electron microscopy encompasses a wide range of microscopy techniques that are employed to identify and characterize EVs derived from biofluids and cell cultures. These are low-throughput techniques, which means they enable the detailed examination of only a few particles at a time. Despite this limitation, they are highly valuable for providing in-depth information regarding the size, shape, and morphology of EVs (68). The majority of electron microscopy (EM) techniques can be classified into scanning electron microscopy (SEM), transmission electron microscopy (TEM), cryogenic electron microscopy (Cryo-EM), and atomic force microscopy (AFM) (69–71). TEM: Provides high-resolution morphological images of EVs (e.g., cup-shaped structures), though sample preparation requiring fixation and dehydration may cause EV deformation (68). Cryo-EM: Observes native EV structures under cryogenic conditions, resolving the bilayer membrane features of exosomes, which is used for morphological validation of immunotherapy-related EVs (e.g., antigen-loaded dendritic cell EVs) (69).Although these EM techniques have demonstrated their utility in confirming the topological features of EVs and other physical characteristics, it is important to note that the observed EVs are often not in their native form. This is because the sample preparation procedures for several EM techniques, such as SEM, TEM, Cryo-TEM, and AFM, subject the EVs to extreme conditions. These conditions include chemical drying, freezing, and layer sectioning, among others. Such treatments can alter the structure of the EVs before they are observed under the microscope. This limitation should be taken into account when selecting an appropriate electron microscopy technique for EV analysis. In addition, all EM techniques share the disadvantage of low sample throughput. This makes it challenging to observe a large number of samples or multiple samples simultaneously.

##### 2.2.2.3 Deeper integration of spectral characterization techniques with microscopic characterization techniques

The more profound integration of spectral characterization techniques with microscopic characterization techniques offers valuable insights into the surface interface properties and enables the characterization of the nanoscale chemical structure of EVs. Currently, there are four novel techniques available: surface-enhanced Raman scattering (SERS) (72), tip enhanced Raman spectroscopy (TERS) (73).Scattering-type Scanning near-field Optical microscopy (S-SNOM) (74) and Atomic Force microscopy-infrared spectroscopy (AFM-IR) (75). Antibody-functionalized SERS has the ability to target specific EVs, and these targeted EVs can then be detected through signal amplification (76). One of the primary advantages of SERS labeling is that, compared to fluorescent labeling, Raman spectroscopy exhibits superior photochemical stability. This is due to the vibrational nature of the signal it generates. This technique has been specifically developed for the detection of EVs in tumors (77). When it comes to the S-SNOM technique, the spectra obtained are prone to shifts in the position of the absorption peaks. Such shifts lead to distortions and artifacts, which are not conducive to revealing the properties of biological specimens and determining protein structures (78). In the case of the TERS technique, the spectra acquired from biological systems generally lack the amide band I. This band is crucial for studying the structure and interactions of proteins (73). These aspects have presented obstacles to the high-throughput application of TERS and S-SNOM for the detection of EVs in clinical settings. For AFM-IR, it can leverage the advantage of AFM, high spatial resolution (1-10 nm), to measure infrared absorption associated with sample thermal expansion. This measurement can be carried out without the need for additional enhancement factors, regardless of the properties of the sample and the AFM probe (75). The advantage of AFM-IR lies in its ability to detect changes in nucleic acids, lipids, and proteins within a small number of EVs. This characteristic endows it with great potential for the early diagnosis of diseases (79).

#### 2.2.3 EVs analysis

**Figure 4:**
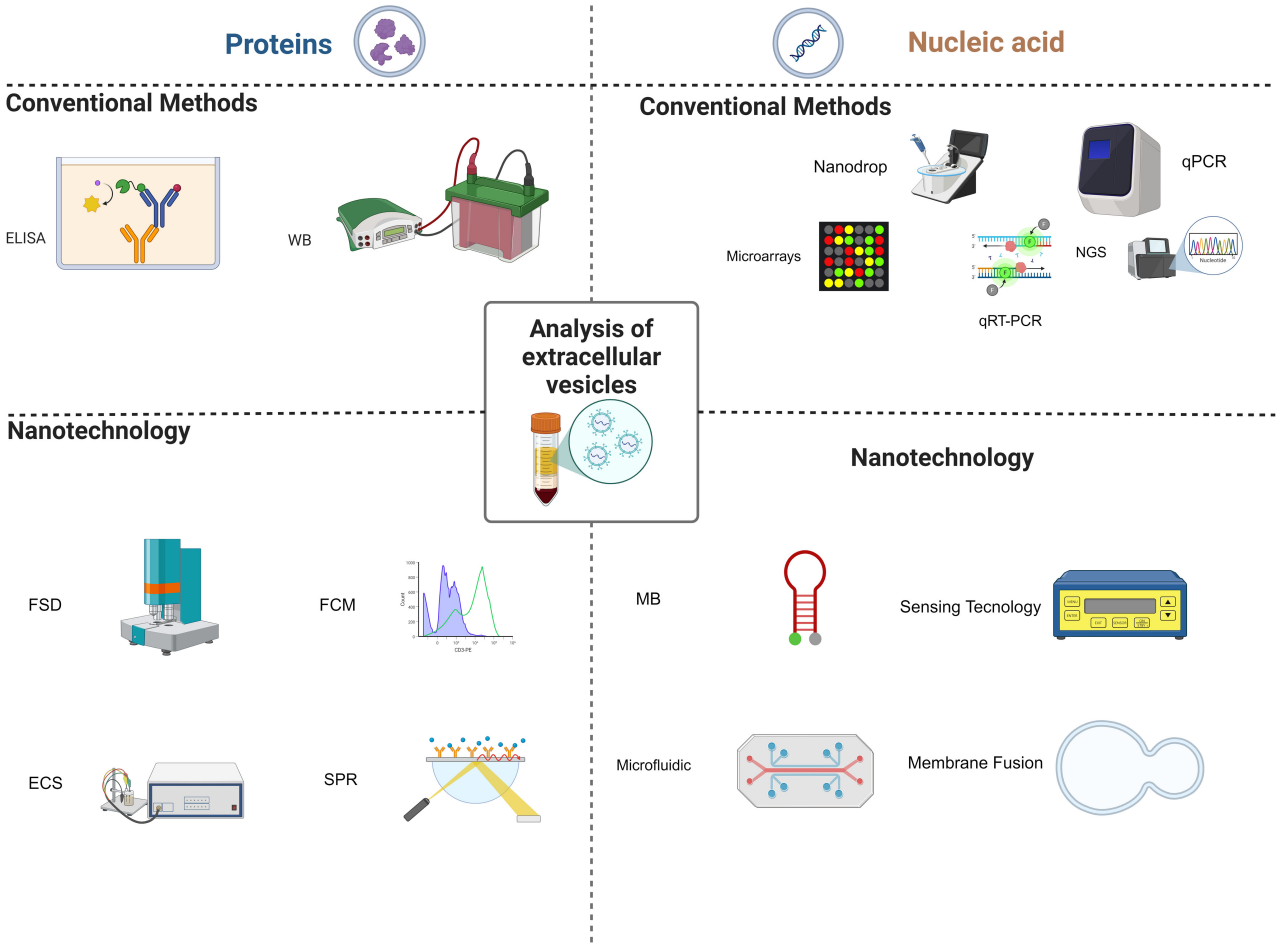
Analysis of extracellular vesicles. Conventional methods for analyzing extracellular vesicles (EVs) include the enzyme-linked immunosorbent assay (ELISA) or Western blot (WB) for detecting EVs-related proteins. For EVs-related nucleic acids, techniques such as NanoDrop for nucleic acid quantification, quantitative reverse transcription PCR (qRT-PCR), and next-generation sequencing (NGS) are commonly employed. Nanotechnology-based methods, on the other hand, involve the use of flow cytometry (FCM), fluorescence detection (FSD), and electrochemical sensors (ECS), as well as surface plasmon resonance (SPR) for the analysis of EVs-related proteins. When it comes to EVs-related nucleic acids, methods like molecular beacons (MB), sensing technologies, microfluidic chips, and membrane fusion techniques are utilized.

The analysis of EVs cargo is of utmost importance for biomarker discovery. Research on EVs cargo associated with predicting the efficacy of cancer immunotherapy primarily centers around proteins and nucleic acids, with miRNAs being a major component. Hence, just as shown in **Figure 4**, this chapter will be dedicated to the analysis methods of EVs-related proteins and nucleic acids.

##### 2.2.3.1 Proteins

Traditional approaches for protein analysis frequently rely on the enzyme-linked immunosorbent assay (ELISA) or Western Blot (WB) to assess EV protein biomarkers. Nevertheless, these methods are marked by low sensitivity and low throughput. Specifically, they can only evaluate a single biomarker or a limited number of biomarkers at one time (80). Given that nanoscale EVs are vulnerable to interference from background noise, effective signal transduction and amplification are indispensable for the detection and molecular analysis of EVs. A variety of detection methods have been developed for EVs detection and molecular analysis, such as colorimetry (81), electrochemical assays (82), surface plasmon resonance (SPR) sensors (83), and so on.

Fluorescence detection (FSD) is one of the most classic methods for detecting EVs. Enriched EVs are commonly stained with fluorescent probes that target membrane proteins or lipids, facilitating their visualization, detection, and molecular analysis. With the advancement of labeling techniques and fluorescent probes, it has become possible to conduct multiple analyses of different markers on EVs, including proteins (84). For instance, by labeling the captured EVs with a lipophilic fluorescent dye and an antibody-coupled quantum dot probe, the fluorescence signal from the quantum dot can be normalized using the signal from the lipophilic dye as a reference, enabling the quantification of the captured EVs (85).

The electrochemical sensor (ECS) is one of the classic sensing systems for EVs detection. It is characterized by high sensitivity, rapid response, portability, and ease of integration with microfluidic chips. Nanomaterials are utilized to coat electrode substrates or serve as reporters, capitalizing on their large surface area and excellent electrical conductivity to enhance signal transduction and amplification (86, 87). A typical example is the integrated magneto electrochemical EVs system, which combines immunomagnetic microbead-based EVs enrichment and electrochemical sensing for EVs detection. Microfluidic platforms have been employed to achieve accurate analyses at the single-particle level through electrochemical detection (88).

Nano plasma sensors based on local surface plasmon resonance (NPS-LSPR) have been widely applied in EVs analysis due to their high sensitivity and colorimetric detection capability. These nano plasma sensors typically feature regularly distributed nanostructures to boost sensitivity and signal amplification. A representative of nanostructure-based nano plasmonic sensors is the nano plasmonic EVs sensor system. This system is based on transmission surface plasmonic resonance and is patterned on a gold film with a thickness close to the size of EVs using an antibody-functionalized periodic nanopore array, thus achieving high sensitivity (89). When EVs bind to the nanopores, it leads to a spectral shift or intensity change that is proportional to the expression level of labeled proteins on the EVs. Signal amplification is accomplished by introducing spherical or star-shaped gold nanoparticles for the secondary labeling of the captured EVs. This sensor system allows for the parallel analysis of up to 12 labeled proteins. Nano plasma enhanced scattering is a method for detecting EVs that is based on the principle that gold nanoparticles of varying sizes and shapes scatter light at characteristic wavelengths. When the designed gold nanospheres and gold nanorods are combined on the same EVs, due to the nanoscale size of the EVs (< 200 nm), their scattering is coupled, generating a local plasma effect that turns the spectrum of the scattered light yellow. This system enables the ultrasensitive detection of EVs from as little as 1 μl of plasma. Surface enhanced Raman spectroscopy sensors have been rapidly adopted for detecting cell-secreted EVs because of their remarkable features, such as non-invasive analysis compared to standard enzyme-linked tests. Typically, the trapping substrate consists of magnetic beads that are further molecularly functionalized for specific EVs binding.

Batch separation, extraction, and analysis of EVs may lead to inaccuracies due to the differences among individual EVs (90). Therefore, several techniques have been developed to address these challenges by analyzing the information of individual EVs. Examples include fluorescence-activated vesicle sorting (91) and high resolution FCM (92), which can more reliably quantify the expression of cancer-related proteins and surface biomarkers compared to traditional flow cytometry (93). Currently, efforts are being made to explore the development of nanoflow cytometry as a liquid biopsy platform for diagnosing cancer biomarkers (94). Other methods for detecting individual small EVs include photoactivation localization microscopy/Random optical reconstruction microscopy with super-resolution microscopy (95), quantitative single-molecule localization microreplication (96), and interparticle iron reflection imaging sensors (97).

##### 2.2.3.2 Nucleic acid

EVs encapsulate a diverse array of nucleic acids within their lipid structures. For the analysis of EVs-related DNA, DNA enzymes are employed to identify the specific forms of DNA fragments that are encapsulated within EVs (98, 99). In terms of quantification, the concentrations of EVs-related nucleic acids can be determined using a NanoDrop instrument, which relies on ultraviolet absorption (100). In contrast, fluorescence quantification, which involves combining DNA with fluorescent dyes, offers greater sensitivity and specificity towards targeted nucleic acids. Additionally, the lengths of EVs-associated DNA fragments can be labeled using a DNA ladder through agarose gel electrophoresis (101, 102). Jiao et al. developed a hydrogel-based droplet digital multiple displacement amplification method, enabling a comprehensive analysis of EVs-related DNA at the level of individual EVs (103).

Techniques such as microarrays, real-time quantitative reverse transcription PCR (qRT-PCR), and next-generation sequencing (NGS) have been extensively utilized to quantify the expression levels of exosomal RNA. However, each of these methods has its own drawbacks, as they are tailored to specific purposes with their unique capabilities (104). The analysis of EVs-related RNA predominantly focuses on microRNAs (miRNAs). A variety of probes have been employed for the detection of miRNAs in EVs, including molecular beacon (MB) probes, self-assembled probes with various structures, and certain nanomaterial probes. An MB is a hairpin-shaped probe designed as a stem-loop structure, with a fluorescent dye and a quencher attached to its two ends. When the MB binds to the target miRNA, the hairpin structure unfolds, separating the fluorescent dye and the quencher spatially, which then allows the fluorescent dye to emit fluorescence. Using this approach, several MBs have been designed for the detection of miRNAs in EVs, such as miRNA-21 (105) and miRNA-375 (106). DNA can be engineered into various elaborate structures. Through precise spatial control, different probes can be anchored in the designated positions, thereby increasing the concentration of probes within a specific space and enhancing the detection efficiency. Due to its relatively large-scale producibility and simple synthesis process, the DNA tetrahedron has become one of the most widely used nanostructures in biomedicine (107). Some nanomaterials have also been utilized to bind nucleic acid probes for the detection of miRNAs in EVs (107). Gold nanoparticles are commonly used nanomaterials, boasting advantages such as good stability, high adsorption capacity for nucleic acid ligands, and ease of synthesis (108).

Fluorescent labeling provides a convenient and visual approach for biomolecular detection and is a classic method for the analysis of EVs-related miRNAs. Nano torches, composed of gold nanoparticles functionalized with fluorescence-labeled single-stranded DNA probes, allow for the direct analysis of RNA within cells or vesicles without the need for cleavage and RNA extraction (81).

Signal amplification strategies for the detection of EVs-related miRNAs mainly include enzymatic and non-enzymatic approaches. Enzyme-assisted amplification is a DNA amplification reaction triggered by different enzymes under isothermal conditions. A dual-signal amplification biosensor was developed for the sensitive detection of EVs-miRNA-21 (109). Catalytic hairpin assembly (CHA) is a common enzyme-free signal amplification method. It relies on a series of primers and two hairpin probes to function. Many studies have combined CHA hairpin probes with DNA nanostructures, as DNA nanostructures can increase the local concentration of hairpin probes in a given space and improve the collision efficiency among the probes (110).

Sensing technology has also been widely applied in the detection of EVs-miRNAs due to its high sensitivity, real-time monitoring capabilities, and flexibility in integrating with other technologies and devices. The sensing methods for detecting EVs-miRNAs mainly include optical sensors, such as SERS and surface plasmon resonance, as well as non-optical sensors, such as electrochemical detection.

Currently, the majority of EVs-miRNA detection methods involve extracting miRNAs after cleaving the EVs. However, once the EVs-miRNAs are separated from the protection of the EVs membrane, they are prone to degradation, leading to insufficient detection sensitivity (111). *In-situ* detection based on membrane fusion represents a novel approach for the *in-situ* detection of miRNAs. By preparing an artificial membrane capsule containing the probe, the probe can be introduced into the EVs without damaging the EVs membrane through membrane fusion. The probe then reacts with the EVs-miRNA and emits signals for the detection of EVs-miRNA (112).

A microfluidic chip, also known as a lab-on-a-chip, is a technology characterized by the manipulation of fluids in a micrometer-scale space (112). Lu et al. designed a portable system for the isothermal amplification and detection of EVs-miRNA. This system consists of two separate chips: one for the enrichment and cleavage of EVs, and the other for miRNA detection. The EVs are first captured using magnetic beads, and then the cleaved EVs release the miRNAs. Subsequently, the EVs are directly transferred to the miRNA detection chip to quantitatively measure the miRNAs carried by specific EVs (112).

In conclusion, the development of numerous new technologies has significantly enhanced the detection efficiency of EVs and their cargo. Nevertheless, the widespread application of nanotechnology-based EVs detection in cancer management has not yet become routine in clinical practice. The relatively limited understanding of the correlation and efficiency of different nanotechnologies in EVs detection has hindered the standardization and industrialization of nanotechnology-based EVs detection. Therefore, it is anticipated that more extensive and in-depth clinical translational research will be carried out in the future.

## 3 The relationship between EVs and tumor immune microenvironment and the detection methods of tumor-related EVs

### 3.1 The relationship between EVs and tumor immune microenvironment

The occurrence and development of tumors are intricately linked to the tumor immune microenvironment. Research has indicated that tumor progression is attributable to immune escape (113–115).Tumor cells can elude immune cells by releasing immunosuppressive molecules or losing adhesion molecules. They can also induce apoptosis through the overexpression of anti-apoptotic molecules or immunosuppressive receptor ligands (116).Moreover, tumor antigens are heterogeneous and have a high mutation rate, rendering it difficult for immune cells to recognize and eliminate tumor cells. EVs, secreted by a diverse range of cell types, play a pivotal role in intercellular signaling. They are increasingly acknowledged as key molecular components in shaping the immunosuppressive microenvironment within the tumor microenvironment. The tumor immune microenvironment harbors a variety of immunosuppressive molecules and cells, which facilitate immune evasion and cancer progression (117).Complex interactions among malignant cells, endothelial cells, stromal cells, and immune cells govern the homeostasis and evolution of the tumor microenvironment. EVs, serving as crucial mediators, play a vital role in intercellular communication by enabling the transfer of cellular components such as lipids, proteins, and nucleic acids between cells (118, 119).Tumor -derived EVs are of great significance in the complex tumor immune network (120).These vesicles possess multiple functions, including regulating tumor growth, promoting neovascularization, enabling immune escape, and facilitating tumor invasion and metastasis (121). Consequently, EVs not only help regulate cell-to-cell communication among cancer cells but also communication among cells within the tumor microenvironment (122).Cancer immunotherapy, which effectively eradicates tumor cells by enhancing the immune system function of cancer patients, has recently emerged as a novel and successful treatment strategy (123).Therefore, EVs hold promise as a biomarker for immunotherapy.

### 3.2 The detection methods of tumor-related EVs

EVs are named differently according to their cell origin. Those derived from tumor cells are termed tumor-associated EVs. Tumor-associated EVs are closely intertwined with the tumor immune microenvironment. As a result, the detection of tumor - associated EVs and their corresponding molecular markers is of particular importance for predicting and evaluating the efficacy of immunotherapy. The current challenge in this area is to establish a “gold standard” for the detection of tumor - associated EVs. This standard should be able to provide morphologically intact, purified, and functional endocytogenic vesicles with high recovery and reproducibility for processing clinical samples. However, EVs in biological fluids are heterogeneous in size, origin, and composition. Moreover, it is possible that tumor-associated EVs share the same physicochemical properties as other EVs. Additionally, the co- separation of EVs with liposomes, chylomicrons, and the presence of contaminants like lipoproteins or protein aggregates pose common obstacles to the currently used separation techniques (124). SEC, a size - based separation technique widely used for high-resolution separation of macromolecules, has been adapted for EVs separation. SEC is especially suitable for isolating EVs from biological liquids as it can remove most, though not all, of the contaminating proteins. SEC offers advantages such as minimal volume requirements, minimal sample loss, and it is a high-throughput, relatively fast method for isolating a high yield of intact, bioactive EVs even with a small sample size (125).Nevertheless, SEC is not without flaws. It fails to successfully separate vesicles from lipoproteins and similarly sized protein aggregates. To eliminate lipoproteins, additional ultrafiltration steps are often necessary (126). Many advanced and complex techniques have been developed to detect EVs or EVs subgroups (90).

For a specific tumor-associated EV, a customized isolation method may need to be designed. Total EVs may first need to be isolated from body fluids and then captured based on the availability of one or a set of antibodies that are overexpressed in parental tumor cells and are specific to the antigen of tumor-associated EVs. There are specific antigens on the surface of tumor-associated EVs, enabling their isolation from non-tumor-derived vesicles through immunoaffinity capture using relevant specific antibodies. Antibody mixtures, such as Epithelial cell adhesion molecule (EPCAM), epidermal growth factor receptor (EGFR), or chondroitin sulfate proteoglycan 4 (CSPG4), have been used to enrich tumor-associated EVs by identifying highly overexpressed tumor-associated EVs on tumor cells and have been applied to construct microarrays for capturing EVs from body fluids (127–129).

Researchers have developed a two- step approach for separating tumor-associated EVs from non-tumor-associated EVs. This approach combines initially purified and enriched SEC with immunoaffinity-based tumor-associated EVs capture, using antigens that are only present in tumors and tumor-associated EVs but not expressed in normal cells or non-tumor-associated EVs (130).Studies have also utilized microarrays or chips coated with antibodies against antigens overexpressed by tumor cells to capture tumor-associated EVs from body fluids. Recently, the above- described immunocapture method has been applied to isolate tumor-associated EVs from the plasma of patients with metastatic melanoma (130). By using the epitope - specific monoclonal antibody CSPG4, melanoma cell-derived EVs can be isolated from other vesicles (non-melanoma-associated EVs) in patient plasma, and the EVs can be analyzed by flow cytometry. The analysis revealed that melanoma-associated antigens such as tyrosinase-associated protein 2 (TYRP2) or MelanA were only carried by melanoma-associated EVs and were not detected in non-melanoma EVs or EVs recovered from the plasma of healthy individuals. Thus, in practice, combining two or more techniques offers the best strategy for isolating tumor-associated EVs.

## 4 Application of EVs as a molecular marker in cancer immunotherapy monitoring

The biochemical characteristics and origin of EVs endow these nanoparticles with great potential as biomarkers in cancer diagnosis, prognosis assessment, and therapeutic monitoring. Moreover, the cargo within EVs, such as RNA, DNA, proteins, and others, are shielded by the natural lipid bilayer capsules from unfavorable biological impacts (like ribonucleases, deoxyribonucleases, proteases) and environmental conditions. As presented in **Table 1**, this chapter provides a review of the applications of EVs cargo as molecular markers in immunotherapy. Since most current studies on the screening of molecular markers of EVs in immunotherapy have focused on NSCLC and melanoma, this chapter primarily focuses on these two cancers.

**Table 1:** Literatures on immunotherapy efficacy prediction of EVs-related molecular profiles.

Molecular profiles of EVs	Body fluid types	Tumor types	Key results	Literature references
miRNA(miR)				
miR-200c-3p,miR-21-5p miR-28-5p	Plasma	NSCLC	Association with poor response. AUC for the combination (miR-21-5p, miR-28-5p and miR-199a-3p)=0.925; AUC for (PD-L1 tissue expression)=0.575	Shukuya et al., 2020 (15)
miR-320d,miR-320c,miR-320b	Plasma	NSCLC	Association with poor response. Association with progressive disease compared to partial response for baseline levels.	Peng et al., 2020 (131)
miR-125b-5p	Plasma	NSCLC	Reduction in miR-125b-5p post-treatment levels when compared to pre-treatment samples among those who achieved a partial response.	Peng et al., 2020 (131)
miR-200c and miR-34a	Plasma	NSCLC	Associated with response and prognosis in patients with advanced NSCLC receiving anti-PD-1 immunotherapy	Monastirioti A,et al.2022 (132)
Circular RNAs				
circCCAR1	Plasma	HCC	Promotes CD8 + T cell dysfunction and anti-PD1 resistance	Hu Z,et al,2023 (133)
circUHRF1	Plasma	HCC	Induces natural killer cell exhaustion and resistance to anti-PD-1 therapy	Zhang PF,et al (134)
circUSP7	Plasma	NSCLC	Induces CD8+ T cell dysfunction and anti-PD1 resistance	Chen SW,et al 2021 (135)
Proteins				
AnnexinA2 S100A8/9	blood	NSCLC	Protein decrease is associated immune response	Brocco D,et al (136)
PD-L1	Plasma	NSCLC	Association with poor response, shorter PFS and OS for the increase in EVs PD-L1following treatment with immunotherapy.	Miguel-Perez et al., 2022 (137)
PD-L1(mRNA)	Plasma	Melanoma and NSCLC	Association with poor response for the increase in EVs PD-L1 following treatment with immunotherapy.	Del Re et al., 2018 (138)
PD-L1	Plasma	Melanoma	Association with poor response for pre-treatment plasma EVs PD-L1 protein levels. Association with improved response for the increase in EVs PD-L1among responders. This observation was not found among non-responders.	Chen et al., 2018 (139)
PD-L1	Plasma	Melanoma	Association with poor response especially in an increase of EVs PD-L1. EVs PD-L1 was detected in all patients (100%) whereas only 67% were PD-L1 positive in tumor biopsies. AUC for Δ PD-L1 = 0.87 for discriminating between responders and non-responders.	Cordonnier et al., 2020 (140)
PD-L1 CD73	Serum	Melanoma	Association with improved response for the increase in EVs PD-L1among responders. Association with poor response for the increase in EVs CD73 among non-responders	Turiello et al., 2022 (141)
EV biomarkers from T-cells (PD-1 and CD28) and dendritic cells (CD80 and CD86) based on fow cytometry analysis	Plasma	Melanoma	Association of baseline EVs PD-1 and CD28 from T cells with improved survival. Upregulated levels of costimulatory molecules (CD80 and CD86) on dendritic cells at the end of immunotherapy treatment in patients who achieved a longer survival.	Tucci et al., 2017 (142)
EV biomarkers from T-cells (PD-1) and melanoma cells		Melanoma	Association of higher levels of PD-1+ EVs from CD8+ T cells with poor response. Association of higher levels of PD-L1+ EVs from melanoma cells with poor response. AUC=0.86 for the combination of (PD-1 EVs from CD8+ T cells and PD-L1+EVs from melanoma cells) showing a strong predictive value for poor prognosis.	Serratì et al., 2022 (143)

AUC, area under curve; EVs, extracellular vesicles; PD-1, programmed death receptor 1; PD-L1, programmed death ligand 1; NSCLC, non-small cell lung cancer; HCC, hepatocellular carcinoma; PFS, progression free survival; OS, overall survival.

### 4.1 RNA

Subsequent research has verified that the RNA carried by EVs participates in intercellular communication (31). Conventionally, the discovery of EVs biomarkers has mainly centered on miRNAs, yet long-stranded RNAs (such as mRNAs, lncRNAs, etc.) carried by EVs are more valuable in detecting somatic mutations and alterations in gene transcription. Among all types of RNA, miRNAs, as predictive biomarkers, have been the most intensively studied in clinical practice. This is attributed to their high abundance, stability, ease of analysis, and their unique function in mediating cell interactions within the tumor microenvironment (28). Studies have reported that in patients with advanced Non-small cell lung cancer (NSCLC) who did not respond to anti-PD-1 or anti-PD-L1 treatment, the levels of miRNA-200c-3p, miRNA-21-5p, and miRNA-28-5p in plasma EVs prior to treatment were elevated (15). Moreover, the combination of three biomarkers, namely miRNA-199a-3p, miRNA-21-5p, and miRNA-28-5p, was more effective in predicting the response to immunotherapy than the PD-L1 expression evaluated through immunohistochemical assessments. miRNAs such as miRNA-320d, miRNA-320c, and miRNA-320b were able to predict the partial responses of advanced NSCLC patients to ICIs (131).Additionally, miRNA-125b-5p, a suppressor of T cells, significantly decreased in the plasma of samples that showed a partial response to ICIs after treatment when compared to the pre-treatment levels (131). Several studies have confirmed the significance of miRNAs as the preferred biomarkers for patients with advanced NSCLC. Notably, three miRNAs from the hsa-miRNA-320 family were identified as potential predictors, and hsa-miRNA-125b-5p was found to be a potential target for anti-PD-1 therapy, as it was downregulated in patients who responded to this treatment. The findings of this study suggest that patients with low levels of miRNA-320d, miRNA-320c, miRNA-320b, and miRNA-125b-5p may be more suitable candidates for anti-PD-1 therapy. A continuous decrease in the levels of the T cell suppressor (miRNA-125b-5p) due to enhanced T cell function can be regarded as an indicator of better treatment outcomes and longer progression-free survival (PFS) (131).miRNA-200c and miRNA-34a in plasma were also associated with the response and prognosis of patients with advanced NSCLC receiving anti-PD1 immunotherapy (132).

In addition to miRNAs, EVs also contain circular RNAs (circRNAs).The latter serve as the foundation for several mechanisms through which they can confer resistance to certain cancer therapies, including immunotherapy. EVs-derived circCCAR1 has been demonstrated to exacerbate CD8+ T cell dysfunction and resistance to anti-PD-1 therapy in liver cancer patients (133).Moreover, cancer-derived EVs-derived circUSP7 may induce CD8+ T cell dysfunction and anti-PD-1 resistance in NSCLC patients by regulating the miR-934/SHP2 axis (135). EVs-derived cirHRF1 is mainly secreted by hepatocellular carcinoma (HCC) cells and exerts an immunosuppressive effect in HCC by inducing natural killer (NK) cell dysfunction. CirHRF1 may contribute to resistance to anti-PD-1 immunotherapy, offering a potential therapeutic strategy for HCC patients (134).

### 4.2 Protein

EVs-related proteins associated with neutrophils (such as annexin A2 and S100A8/9) decreased during treatment with ICIs, whereas positive changes were noted in patients who did not respond to the treatment (136). In line with these findings, a recent study has associated adverse reactions to ICIs in NSCLC patients with alterations in plasma proteins related to neutrophil function during the course of treatment (144).

Recent investigations into the dynamics of EVs protein biomarkers in NSCLC have revealed that an increase in EVs PD-L1 levels after ICIs treatment is associated with adverse reactions and poor survival outcomes (137). In melanoma, the dynamic changes in PD-L1 levels have been the focal point of EVs-related RNA and protein analyses, and these changes are linked to the response to ICIs (145). By assessing the PD-L1 mRNA expression in plasma-derived EVs to monitor the treatment response in melanoma and non-small cell lung cancer, a study reported that PD-L1 levels decreased in patients who achieved partial or complete responses, while PD-L1 expression increased in patients who did not respond after ICI treatment (138). Recent studies have indicated that the expression levels of EVs PD-L1 in plasma are significantly higher before treatment in metastatic melanoma patients who do not respond to ICIs (139). Conversely, according to Chen et al., elevated EVs PD-L1 expression levels during the early stage of immunotherapy predicted higher response rates in melanoma patients. Interestingly, this correlation was not observed in non-responders. Thus, the findings of this study suggest that EVs PD-L1 may have different clinical implications depending on factors such as the sampling time, disease duration, treatment planning rules, and the differences between responders and non-responders (139). In this regard, the authors suggest that high levels of EVs PD-L1 at baseline may indicate T cell dysfunction, and that the increase in EVs PD-L1 levels after immunotherapy may be associated with T cell reactivation and an enhanced anti-tumor immune response, which is more evident in responders compared to non-responders whose T cells cannot be restored by immunotherapy. The responders have “less depleted” immunity against the original tumor (139). Additionally, although EVs PD-L1 was detected in all patients in this study, only 67% of patients with tumor biopsies showed positive PD-L1 expression (140). These results emphasize the limitations of PD-L1 in immunohistochemistry as a biomarker for predicting the response to immunotherapy (146), and also support further exploration of EVs PD-L1 in plasma as a promising predictive biomarker in clinical practice.

**Figure 5:**
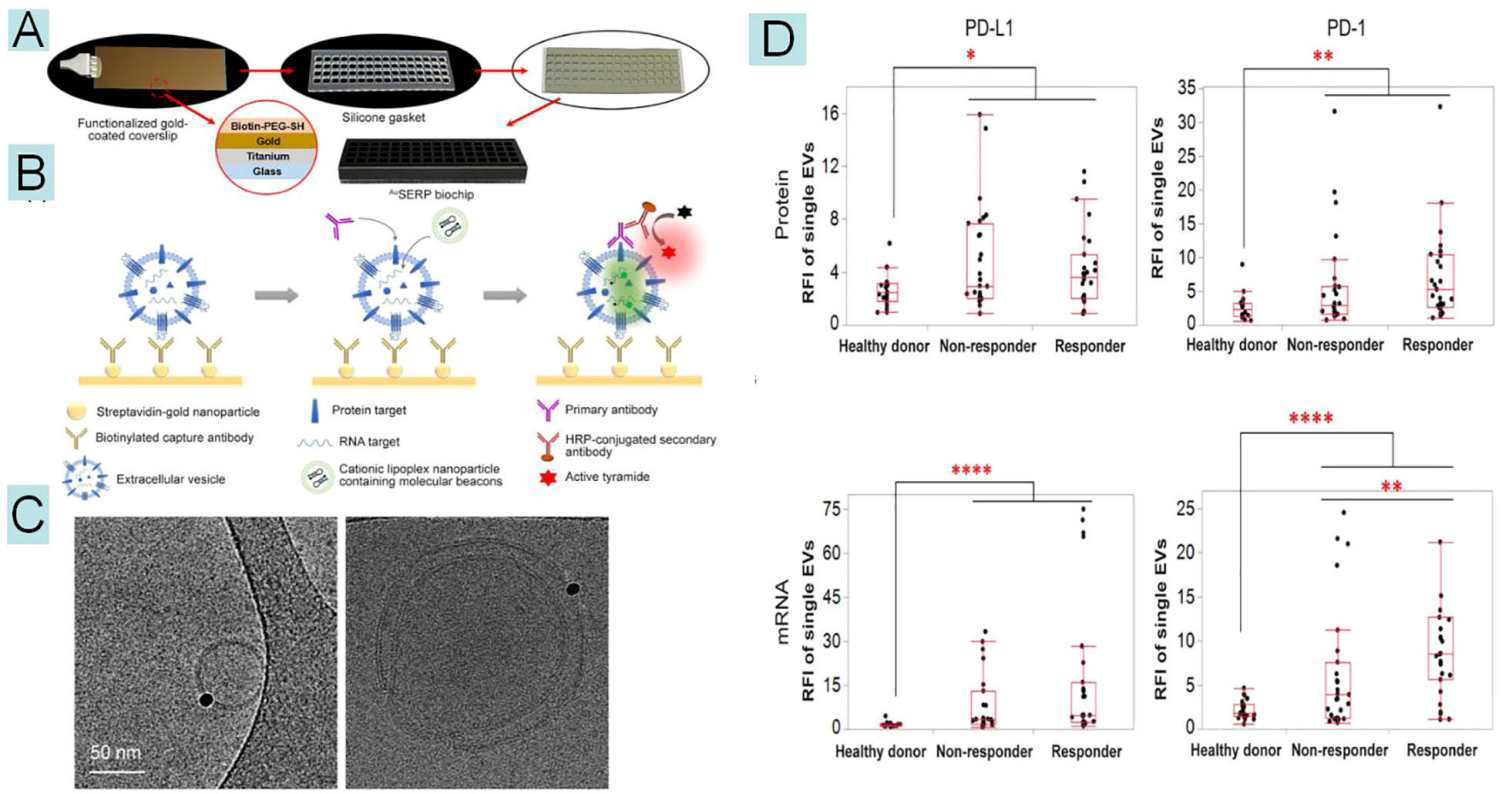
These figures are cited from the research of Nguyen, et al (147). A single extracellular vesicle related RNA and protein(^Au^SERP) assembly. A functionalized gold-coated coverslip was attached to a silicone gasket with 64 chambers for high-throughput analysis of single-EV biomarkers. B.A schematic representation of the mechanism of detection for protein and mRNA biomarkers present in single extracellular vesicles (EVs) using ^Au^SERP. A gold-coated coverslip with PEG-tethered gold nanoparticles (NPs) conjugated to capture antibodies was used to immobilize single EVs. Proteins on the surface of the single EVs were detected using the corresponding primary antibody and a tyramide signal amplification (TSA) method, resulting in fluorescent signals. mRNA was identified using target-specific molecular beacons (MBs) encapsulated in cationic lipoplex nanoparticles (CLNs), resulting in fluorescent signals.C Cryogenic electron microscopy (Cryo-TEM) images of immunogold labelled PD-L1 protein on the EVs surface.D Box plots of quantitative fluorescence intensities of PD-1/PD-L1 protein and mRNA expression levels. 54 patients were evaluated (27 responders and 27 non-responders), along with 20 healthy donors. RFI, relative fluorescence intensity.

EVs released from immune cells have also been reported as a potential source of biomarkers related to the response to ICIs. The study found that in metastatic melanoma patients treated with PD-1 inhibitors, higher levels of PD-1+ EVs, especially those derived from CD8+ T cells, were strongly associated with poorer progression-free survival (143). However, further research is required to better define the predictive value of EV PD-1 in various immunotherapy regimens. Integrating these results with recent efforts to study the dynamics of other immune cells in the blood may lead to the development of a more accurate predictive model for the response to immunotherapy. As shown in **Figure 5**, Luong T. H. Nguyen, et al. (147). proposed an immunogold biochip for quantifying single EVs-related RNA and protein. Using only 20 μl of purified serum, the PD-1/PD-L1 proteins on the surface of EVs and the PD-1/PD-L1 mRNA within EVs were detected at the single-vesicle resolution, with a sensitivity 1000 times higher than that of conventional batch analysis methods such as ELISA and qRT-PCR. A cohort test involving 27 non-responsive and 27 responsive NSCLC patients demonstrated the potential of this method to enhance the prediction of immunotherapy and cancer diagnosis in a clinical setting.

### 4.3 Others

Interestingly, EVs-related lipids can also be relevant to cancer immunotherapy. For instance, EVs associated with B-cell lymphomas have been shown to contain the phosphatidylcholine transporter. This exosome may contribute to immunotherapy resistance by protecting target cells from treatment with rituximab, an antibody that targets the B-cell lymphocyte antigen CD20 (148).

**Figure 5** demonstrates the use of a nanoplatform for detecting EV molecules to assess the efficacy of immunotherapy, which is a typical representation. Currently, based on existing research, most molecular characterizations of EVs still rely on conventional methods to predict immunotherapy efficacy. Therefore, more future research should focus on how to apply nanotechnology for EV detection more extensively in immunotherapy.

## 5 Conclusion

Liquid biopsy is a minimally invasive approach that offers the advantage of real-time monitoring. During the process of immunotherapy, utilizing liquid biopsy technology to analyze and assess the microscopic changes in patients after drug administration is beneficial for determining whether the tumor is progressing. EVs represent a novel method within the realm of liquid biopsy. Additionally, EVs contain a diverse range of biomolecules, predominantly miRNAs and proteins. These biomolecules reflect the heterogeneity of the parental cells and serve as important sources of biomarkers yet to be fully explored. As a crucial medium for intercellular communication in the tumor microenvironment, EVs are inevitably associated with the monitoring of the tumor’s response to immunotherapy. However, detecting and analyzing EVs pose significant technical challenges due to their minute size, heterogeneity, and the difficulty of separating them from complex samples. This is particularly the case because complex components such as proteins, lipoproteins, and lipids are abundantly present in serum or plasma. Consequently, distinguishing EVs from a large number of blood cells and other intricate components in the blood demands high sensitivity and specificity. In this context, nanotechnologies possess remarkable advantages in the detection of EVs. Nonetheless, the extensive application of nanotechnology-based EVs detection in cancer management has not yet been integrated into routine clinical practice. Hence, it is anticipated that more comprehensive clinical translational research will be carried out in the future. Moreover, the molecular markers currently investigated in clinical practice mainly center around EVs-derived miRNAs and PD-L1. Therefore, it is hoped that more studies will be conducted in the future to explore novel molecular markers, which will enable a more accurate detection of the efficacy of immunotherapy.
